# Co-Microencapsulation of Anthocyanins from Cornelian Cherry (*Cornus mas* L.) Fruits and Lactic Acid Bacteria into Antioxidant and Anti-Proliferative Derivative Powders

**DOI:** 10.3390/nu14173458

**Published:** 2022-08-23

**Authors:** Iuliana Maria Enache, Mihaela Aida Vasile, Oana Crăciunescu, Ana Maria Prelipcean, Anca Oancea, Elena Enachi, Viorica Vasilica Barbu, Nicoleta Stănciuc, Camelia Vizireanu

**Affiliations:** 1 Faculty of Food Science and Engineering, Dunarea de Jos University of Galati, 800008 Galati, Romania; 2 National Institute for Research and Development of Biological Sciences in Romania, 060031 Bucharest, Romania

**Keywords:** *Cornus mas*, anthocyanins, *Lacticaseibacillus casei* (*L. casei*), co-microencapsulation, anti-proliferative effect

## Abstract

Driven by the modern lifestyle, the consumers are interested in healthier and balanced diets, including both probiotics and natural antioxidants. The beneficial health effects of probiotics are mainly due to their capacity of modulating the human intestinal microbiota, although achieving at least a 6 log of viable cells at the targeted site is still challenging. Therefore, in this study, an attempt to improve the bioaccessibility of *Lacticaseibacillus casei* (*L. casei*) 431^®^ through a co-microencapsulation by complex coacervation and freeze-drying, using an extract from Cornelian cherry and two biopolymeric matrices, whey protein isolates and casein (WPI-CN) and inulin (WPI-I), was studied. The powders showed a comparable anthocyanin content of around 32.00 mg cyanidin-3-rutinoside (C3R)/g dry matter (DM) and a microbial load of about 10 Log CFU/g DM. A high stability of the lactic acid bacteria was assessed throughout 90 days of storage, whereas the anthocyanins’ degradation during storage followed a first order kinetic model, allowing the estimation of a half-time of 66.88 ± 1.67 days for WPI-CN and 83.60 ± 2.46 days for WPI-I. The in vitro digestion showed a high stability of anthocyanins in the simulated gastric juice, whereas the release in the simulated intestinal juice was favored in the variant with inulin (up to 38%). The use of casein permitted to obtain finer spherosomes, with smaller diameters, whereas a double encapsulation was obvious in both variants, thus explaining the high resistance in the gastric environment. The anti-proliferative effect against the human colon cancer cell line (HT-29) was also demonstrated. No cytotoxicity has been found for the concentrations between 1 and 25 μg/mL for the WPI-I variant, whereas a cell proliferation effect was observed at low concentrations of 1–5 μg/mL.

## 1. Introduction

Food coloring agents reached a worldwide turnover of 8000 tons per year, as they are used in different food applications, such as carbonated drinks, salads, juices, ice creams, and sweets [1]. Food color is a decisive factor in the consumer’s choice; therefore, an estimation in this regard suggested that 62–90% of consumer’s assessment is based on color evaluation [2]. Driven by the consumer’s preferences, which turns the activity into an economically profitable one, the producers are focused on identifying natural alternatives to prevent and eliminate the shortcomings of synthetic dyes, especially those related to possible negative side effects on health, such as obesity, cancer, diabetes, hypertension, dementia, or eye diseases [3]. Several criteria are used for the classification of different natural dyes, such as their origin (vegetal, animal, bacterial, fungal, etc.) or their hue (red, yellow, purple, blue, green, etc.), while the flavonoids (anthocyanins), isoprenoids (carotenoids), and nitrogen-heterocyclic derivatives (betalains) are the most used natural dyes, in a chemical structure’s dependent manner [2]. When considering the long-term use of natural colorants, some significant benefits in the human’s health should be considered, such as increasing the resistance of low-density lipoproteins to lipid peroxidation, the protection of proteins against oxidation, the chelation of transition metals that promote oxidative reactions, and the inhibition of the enzymes involved in the oxidative stress [4].

Among all the natural sources, plants, mainly fruits and vegetables, are naturally rich sources of pigments, with particular characteristics, such as solubility in water or lipids and solvents, significantly different both in structure and metabolic pathways [5]. However, when considering the use of natural pigments, some crucial steps should be considered, such as extraction methods to obtain a maximum extraction yield, purification, and stabilization to provide an appropriate environment for the bioactive to limit the prone to degrade at different parameters. It is well known that bioactives are prone to degradation to light, heat, oxygen, and temperature and can interact with each other or with different components of the food matrix, via polymerization or co-pigmentation reactions. Another critical step in the use of natural pigments is the low bioavailability, which has a crucial importance in biological activity maintenance [6]. However, the remarkable progress in the use of encapsulation technologies, both at scientific and application levels, provides an immense opportunity for industry to provide stable, health-promoting compounds for different fields [7]. The need for natural colorant encapsulation is given by the significant technological and biological advantages, related to their easier handling, increased chemical and thermal stability, preservation, or masking of their aroma, taste and/or scent, controlled and targeted release, and increased bioavailability [7]. In order to respond to the complex demands of the industry, there is a need to develop technological alternatives that address the multifunctional properties of an ingredient by incorporating probiotics [8]. The use of probiotics in food and nutraceuticals is limited due to their low resistance to temperature, light, or oxygen, while their ability to remain metabolically active under the gastrointestinal conditions so as to exert their beneficial effects are still challenging [9]. Numerous studies revealed the prebiotic function of anthocyanins, due to their ability to improve the diversity of the intestinal microbiota [10,11], by different mechanisms, hence allowing the growth of beneficial bacteria and inhibiting potentially harmful ones, and increasing the concentration of short-chain fatty acids [10,12].

Cornelian cherries (*Cornus mas* L.) are well known for their rich content in bioactive compounds, including organic molecules, carbohydrates, fatty acids, vitamins, and minerals [13]. The organic molecules can be divided into five structural groups: anthocyanins, iridoids, phenolic acids, flavonoids, and tannins [14]. The phytochemical profile of Cornelian cherry makes the fruits a purposive food in the food industry [15], being used to produce different drinks, syrups, gels, jams, and other traditional products [16].

The *Lacticaseibacillus casei* (*L. casei*) group is represented by *L. casei*, *L. paracasei*, and *L. rhamnosus* species, being some of the most well studied and applied probiotic species of lactobacilli, especially due to their commercial, industrial, and applied health-promoting potential [17]. Usually, *L. casei* is used in the milk fermentation processes, as starter or adjunct culture, to deliver yogurt or cheeses, and it has been associated with important health-promoting benefits, such as regulation of intestinal microbiota [18], tumor inhibition, pro-apoptotic and anti-proliferative effects [19], and with the production of bioactive peptides in fermented milk [20]. *L. casei* 431^®^ is a probiotic strain associated with several health benefits, including boosting the immune system’s health, maintaining a healthy respiratory tract and bowel function. *L. casei* 431^®^ shows a high stability being able to survive under the gastrointestinal tract and it has been successfully used in food and dietary supplements since 1995 [21].

The aim of our study was to design and test two synbiotic co-microencapsulated delivery systems, containing bioactive compounds from Cornelian cherry (CM) and *L. casei* 431^®^ into biopolymeric whey protein isolated-based materials, including casein and inulin, through complex coacervation and freeze-drying in order to develop potential candidates for food and pharmaceuticals applications. The extract was obtained from freeze-dried CM fruits using a liquid-solid ultrasound-assisted extraction and co-microencapsulated in whey protein isolate-casein (WPI-CN) and whey protein isolate-inulin (WPI-I) with *L. casei* 431^®^ by complex coacervation and freeze-drying. The two biopolymeric combinations were selected based on their properties, in order to increase the co-microencapsulation efficiencies, both for polyphenolic compounds and probiotic cells and to create a protective environment able to allow a good resistance of co-microencapsulated bacteria in the gastrointestinal stress and a controlled release of anthocyanins. The selected microencapsulation matrices have unique properties, both from technological and nutritional points of view. For example, milk proteins are rich sources of essential amino acids and have excellent water binding, gelling, emulsifying, and foaming properties. Moreover, both whey proteins and casein have slow digestion patterns and immune benefits [22,23]. Inulin, a natural dietary soluble fiber consisting of a mixture of oligo- and/or polysaccharides of β (2→1) linked D-fructose units with a terminal glucose residue linked by α (1→2) bond [19], is frequently used as ingredient for functional food, given its prebiotic potential, technological properties, and beneficial effects on health [24].

Therefore, the two powders were tested for anthocyanins and lactic acid bacteria encapsulation efficiency, phytochemical profile, in terms of total polyphenolic content (TPC), total flavonoids content (TFC), total monomeric anthocyanin (TAC), antioxidant activity, cells viability, color, structure and morphology, in vitro bioavailability of anthocyanins, and storage stability. The powders were also tested for anti-proliferative activity on HT-29 human colon cancer cell line and cyto-compatibility on L929 fibroblasts cells.

The obtained results highlighted the effectiveness of the customized co-microencapsulation designs, in terms of polyphenolic compounds, whey proteins, casein, and inulin in preserving the bioactive chemical stability and cells viability during shelf life, whereas a controlled release of anthocyanins in gastrointestinal environment was suggested.

## 2. Materials and Methods

### 2.1. Chemicals

Acetic acid, hydrochloric acid, aluminum chloride, 2,2-Diphenyl-1-picrylhydrazyl (DPPH), ethanol, Folin–Ciocâlteu reagent, gallic acid, inulin from chicory, methanol, pancreatin (Kreon), pepsin from gastric porcine, potassium acetate, Trizma hydrochloride, 6-Hydroxy-2,5,7,8-tetramethylchromane-2-carboxylic acid (Trolox), and inulin were purchased from Sigma Aldrich (Taufkirchen, Germany), while sodium bicarbonate was purchased from Honeywell, Fluka (Selze, Germany) and whey protein isolate 894 (WPI) from Fonterra (Clandeboye, New Zealand). *L. casei* 431^®^ strain was purchased from Chr. Hansen (Hoersholm, Denmark). For the cell viability studies of the *L. casei* 431^®^, de Man, Rogosa, and Sharpe agar (MRS agar) (Merck (Darmstadt, Germany) was used. The human colon cancer cell line (HT-29, American Type Culture Collection—ATCC), fibroblasts from the NCTC cell line clone L929 (European Collection of Authenticated Cell Cultures, Sigma-Aldrich, Darmstadt, Germany), Dulbecco’s Modified Eagle Medium (DMEM), fetal calf serum (FCS), Medium Minim Essential (MEM), L-glutamine, and a mixture of antibiotics (penicillin-streptomycin-neomycin) were used for antiproliferative activity and cytotoxicity.

### 2.2. Fresh Fruits Processing

CM fruits were purchased from a local market (Galati, Romania) in October 2018. The fresh fruits were manually removed from their seeds, washed, and dried with a paper towel and freeze-dried at −42 °C under a pressure of 10 Pa for 48 h. The freeze-dried CM fruits were collected and packed in dark glass containers, hermetically sealed, and stored at 4 °C until analysis.

### 2.3. Phytochemical Extractions

The anthocyanins’ extraction was performed by liquid-solid ultrasonic-assisted method, using ethanol solution as a solvent (70%). An amount of 50 g of freeze-dried CM ground powder was mixed with 400 mL of ethanol solution, followed by ultrasound-assisted extraction at 40 °C for 30 min (100 W and 40 KHz). The resulting liquid containing anthocyanins was centrifuged at 6000× *g* for 20 min and concentrated under reduced pressure at 40 °C (AVC 2-18, Christ, UK) to remove the solvent.

### 2.4. Co-Microencapsulation of the Anthocyanins and Lactic Acid Bacteria

A volume of 100 mL of biopolymer solutions, obtained by dissolving WPI and CN (WPI-CN) and WPI and I (WPI-I), in a ratio of 1:1 (*w*:*w*) was mixed with the extract in a ratio of 2.5%. The solutions were allowed to mix on a magnetic stirrer until complete hydration at 650 rpm, and the pH was adjusted at 4.6. The inoculation of the mixture with *L. casei* 431^®^ lyophilized culture (with an initial CFU of 10^11^/g) was performed as described by Enache et al. [25]. The resulting freeze-dried powders were collected, packed in metallized bags, and kept at 4 °C until further analysis.

### 2.5. Phytochemical Profile of the Extract and Freeze-Dried Powders

The extract and freeze-dried powders were analyzed for TPC by Folin–Ciocalteu method, TAC using pH differential method, TFC by aluminum chloride method, and antioxidant activity using DPPH method, as previously described [25]. Briefly, for TPC evaluation, a volume of 0.2 mL of corresponding extract was diluted with 15.8 mL of distillated water, followed by addition of 1 mL of Folin–Ciocalteu reagent and 3 mL of 20% of Na_2_CO_3_. The mixtures were vigorously agitated and allowed to react for 60 min in the dark, followed by the absorbance reading at λ = 765 nm (Jenway Scientific Instruments, Essex, UK) The TPC was expressed as mg gallic acid equivalents (mg GAE) per g dry matter (DM extract or powder), using a gallic acid calibration curve.

For TFC evaluation, a volume of 0.250 mL of the extract or powder extract was mixed with 0.075 mL of sodium nitrite (5%), 0.15 mL aluminum chloride (10%), and 0.5 mL sodium hydroxide (1 M). The absorbance of the mixture was measured at 510 nm against the suitable blank. The TF was expressed in mg catechin equivalents (CE) per g of DM (extract or powder), using a catechin calibration curve.

The DPPH antiradical scavenging potential, a volume of 3.9 mL of DPPH solution (0.1 M in methanol) and 0.1 mL of the extract and powders extract were thoroughly mixed and allowed to react for 30 min, in the dark. The absorbance was read at the wavelength of 515 nm, using a blank with methanol instead of sample. The scavenging percentage of DPPH was expressed as mMol Trolox/g DM using a calibration curve.

### 2.6. Viability of L. casei 431^®^

Sterile physiological serum (0.9 g NaCl%, *w*/*v*) and the pour plate technique were used to estimate the number of viable cells of *L. casei* 431^®^. The standardized method [26] was used, involving the estimation of the viable cell number, by the number of colony-forming units (CFU) when cultivated on the MRS-agar plates (medium at pH 5.7) for 48 h of aerobic incubation at 37 °C. The counts were expressed as CFU/g DM.

### 2.7. Co-Microencapsulation Efficiency

The co-microencapsulation efficiency of the anthocyanins was estimated as described earlier by Enache et al. [25]. The method involves two steps for the evaluation of the surface anthocyanins content (*SAC*) and total anthocyanins content (*TAC*). Two different medium were used to dissolve the powders, namely methanol:acetic acid:distillated water mixture (25:4:21, *v/v/v*) for *TAC* and methanol:ethanol in a ratio of 1:1 (*v/v*) for *SAC*. An amount of 0.2 g of co-microencapsulated powder was dissolved in 5 mL of appropriate mixture, with and without homogenization, and sonicated to destroy the microparticles for *TAC* and *SAC*, respectively. The total monomeric anthocyanin content in the resulting medium was determined by the pH differential method [27], whereas the results were expressed as mg of cyanidin-3-rutinoside equivalents (C3R) per g of DM of powder (mg/g DM). The co-microencapsulation efficiency (*cME*) was calculated using Equation (1):

(1) $$cME\left(\%\right)=\frac{TAC-SAC}{TAC}\times 100$$

For lactic acid bacteria co-microencapsulation efficiency, the protocol described by Colín-Cruz et al. [28] was used. In brief, the quantification of the viable bacteria was performed by pour plate technique. In order to determine the co-microencapsulation efficiency (%), Equation (2) was used:

(2) $$EE\left(\%\right)=\frac{log\;\left(N\right)}{log\;(N_{0})}$$

where *N* is the number of viable cells (CFU/g) in the powder, and *N*_0_ is the number of viable cells in the mixture before freeze-drying [25].

### 2.8. Structure and Morphology of the Powders

The structure and morphology of the powders were measured using a LSM 710 Carl Zeiss Confocal Laser scanning microscope (Carl Zeiss, Oberkohen, Germany), by using four types of lasers, such as a diode laser (405 nm), Ar-laser (458, 488, 514 nm), DPSS laser (diode pumped solid state—561 nm), and HeNe laser (633 nm). The images were captured and rendered with the black edition of the ZEN 2012 SP1 software (Carl Zeiss, Oberkohen, Germany). The powders’ fluorescence was assessed both in their unlabeled (native) and labeled with the Red Congo (40 μM) fluorophore.

### 2.9. In Vitro Simulated Digestion of Anthocyanins

In vitro digestion of the anthocyanins from the powders was performed using a static digestion model, which involves the addition of the powders in simulated gastric juice (SGJ) at pH 2.0 and simulated intestinal juice (SIJ) at pH 7.7, as described earlier by Enache et al. [25].

### 2.10. Colorimetric Analysis

A CR 410 Chroma Meter (Konica Minolta, Tokyo, Japan) colorimeter was used to appreciate the color of the co-microencapsulated powders. The three coordinates, respectively: L* (brightness, 0 for blackness, 100 for whiteness), a* (positive value for redness, negative value for greenness), and b* (positive value for yellowness, negative value for blueness) were used in this study.

### 2.11. Anti-Proliferative Activity and Cytocompatibility of the Powders

#### 2.11.1. Cell Culture and Treatment

The anti-proliferative activity of the powders was tested on human colon cancer cell line HT-29 (ATCC), whereas the cyto-compatibility was tested on mouse fibroblasts from NCTC clone L929 cell line (ECACC). For the estimation of anti-proliferative activity, the cells were cultured in Dulbecco’s Modified Eagle Medium (DMEM) supplemented with 20% (*v/v*) fetal calf serum (FCS), 2 mM L-glutamine and 1% (*v/v*) antibiotic mixture (penicillin-streptomycin-neomycin), in a humidified atmosphere with 5% CO_2_, at 37 °C, until sub-confluence. For the cyto-compatibility tests, the cell was cultivated on Minimum Essential Medium (MEM) supplemented with 10% (*v/v*) fetal calf serum (FCS), 2 mM L-glutamine, and 1% (*v/v*) antibiotic mixture (penicillin-streptomycin-neomycin) in a humidified atmosphere with 5% CO_2_, at 37 °C, until sub-confluence. For testing the effect of the co-microencapsulated powders, serial dilutions of stock a solution (1 mg/mL) were used, followed by sterile filtration using 0.22 µm membrane filters (Millipore, Burlington, MA, USA). For both experiments, the cells were seeded in 24-well microplates, at a density of 4 × 10^4^ cells/mL, followed by an incubation at 37 °C, in a humidified atmosphere with 5% CO_2_, for 24 h. Further, the culture medium was replaced with fresh medium, containing different concentrations of samples ranging from 10 to 1000 μg/mL and incubated at 37 °C under standard conditions, for 24 h and 48 h, respectively. The cells incubated in the culture medium without the sample were used as the control culture.

#### 2.11.2. Cells’ Viability

For the cells’ viability estimation, the Neutral Red (NR) assay was used, as previously described [29]. Briefly, the method involved the removing the specific culture medium at the end of each incubation period, followed by addition of 50 μg/mL NR solution and incubation at 37 °C, for 3 h. After cell washing, the incorporated dye was released in 1% (*v/v*) acetic acid solution in 50% (*v*/*v*) ethanol by gentle shaking, for 15 min. The number of viable cells was directly proportional with the amount of dye taken up. The optical density was measured at 540 nm in a Sunrise microplate reader (Tecan, Grödig, Austria). The results were reported as percentage relative to the control culture, considered 100% viable and the effective concentration was calculated as the sample concentration that decreased the cell viability down to 50% (EC50).

#### 2.11.3. Cell Morphology

Prior to imaging, the cultured cells were washed, fixed with methanol, and Giemsa stained. The cell morphology of the cells incubated in the presence of the samples was observed by light microscopy, after 48 h of cultivation. The micrographs were acquired with an optical microscope Axio Observer D1 equipped with digital camera (Carl Zeiss, Germany).

### 2.12. Storage Stability

The stability of the phytochemicals and lactic acid bacteria cells in the co-microencapsulated powders was tested after 21 and 90 days of storage in the absence of light at 4 °C.

### 2.13. Kinetics of Anthocyanins and Antioxidant Activity Degradation during Storage

The degradation kinetics of anthocyanin and antioxidant activity during storage was obtained by first order kinetic model, as described by Azarpazhooh et al. [30], by estimating the rate constant against the time. The half-life (*t*_1/2_) values were calculated using Equations (3) and (4).

(3) $$C_{t}=C_{0}exp\left(kt\right)$$

(4) $$t_{1/2}=-\frac{ln\;0.5}{k}$$

where, *C_0_* is the initial anthocyanin contents and *C_t_* is the anthocyanin contents after time *t* (days), while *k* is the first-order kinetic constant [30].

### 2.14. Statistical Analysis

Unless otherwise stated, the data reported in this study represent the average of triplicate analyses and were reported as mean ± standard deviation (SD). The statistical analysis was performed on control-sample pairs using two-paired, two-sample equal variance Student’s *t*-test. Significant differences were considered at *p* < 0.05.

## 3. Results

### 3.1. Phytochemical Characterization of the Extract

The combined liquid-solid solvent-ultrasound assisted extraction method allowed to obtain an extract with TAC of 34.01 ± 2.66 mg C3R/g DM, TPC of 27.00 ± 0.46 mg GAE/g DM, and TFC of 14.31 ± 1.70 mg CE/g DM, leading to an antioxidant activity of 178.43 ± 4.34 mMol/g DW. Gąstoł et al. [31] reported a total polyphenolic CM fruit content of 45.6 mg GAE/g fresh weight (FW), suggesting a higher polyphenols level compared to apple, pear, and plum fruits. Our results for TPC are higher than those reported by Okan et al. [32], who suggested a concentration of 19.87 ± 0.27 mg GAE/g DM in CM fruits. A high level of TAC was found in our study, related to the color of the fruits, when compared to the data from literature. For example, Dzydzan et al. [33] reported that the red CM fruits contain anthocyanins in a concentration of 20.67 mg/g DM. TFC values varied between 0.5 and 207 mg quercetin equivalents (QE)/g DM for different varieties cultivated in Turkey [33]. However, the extraction efficiency of the bioactive from different matrices is highly affected by the extraction method. Therefore, Kutlu et al. [34] used microwave and ohmic heating-assisted microwave methods to extract the phenolic compounds from Cornelian cherry, suggesting that a preliminary ohmic treatment prior to the extraction enhanced the TPC yield 1.1- and 5.4-fold compared to that of the microwave and maceration, respectively. These authors reported a lower TAC value of 0.65 mg C3G/g DM, whereas a comparable value for the TPC was suggested (29.4 ± 0.67 mg GAE/g DM) by using a combination of ohmic heating assisted with microwave extraction.

### 3.2. Co-Microencapsulation Efficiency and Phytochemical Profile of the Powders

In terms of the co-microencapsulation efficiency of anthocyanins, values of 77.97 ± 0.57% for WPI-CN and 79.03 ± 0.72% for WPI-I were determined using Equation (1). The co-microencapsulation efficiency of the lactic acid bacteria was of 90% for both of the powders. The phytochemical profiles of the powders are given in Table 1.

It can be observed that no significant differences were found in the bioactive content between the two co-microencapsulation matrices, whereas WPI-CN revealed a higher antioxidant activity. Oancea et al. [35] microencapsulated anthocyanins from cherry skins in whey protein isolate and reported an encapsulation efficiency of 70.30 ± 2.20%, while Tao et al. [36] suggested that the encapsulation efficiency of blueberry anthocyanins using different ratios of WPI, acacia gum, maltodextrin, and β-cyclodextrin reached 82%. However, the biopolymeric combination used in our study allowed to enhance the microencapsulation efficiency of lactic acid bacteria. Neuenfeldt et al. [8] suggested values ranging between 82 and 84% for *L. rhamnosus* co-microencapsulation with blueberry extract and Arabic gum, inulin, and maltodextrin.

### 3.3. Structural and Morphological Analysis of the Powders

**Figure 1:**
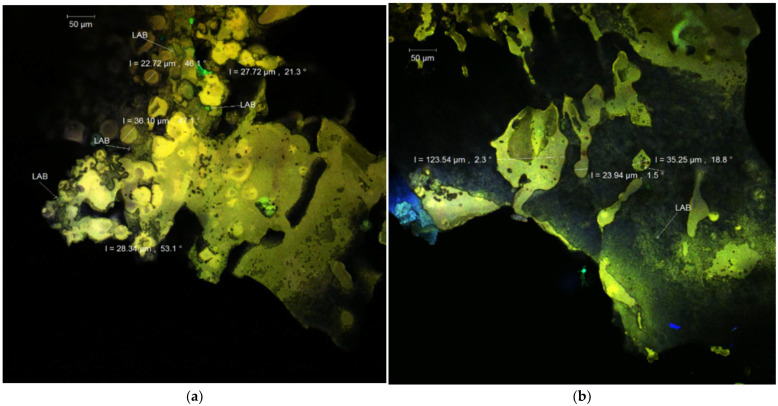
Confocal laser scanning microscopy images of the co-microencapsulated extract from Cornelian cherry fruits with *L. casei* 431^®^ in whey protein isolates and casein (**a**) and whey protein isolates and inulin (**b**) in native state.

The native samples of powder (Figure 1a,b) revealed irregular, polygonal, large, and thin formations with autofluorescence between 500 and 540 nm, in which anthocyanins and lactic acid bacteria are trapped in the matrix network. If in the WPI-I powder, the spherosomes are scarce and the scale-like formations are predominant (Figure 1b); when CN was used as an encapsulating adjuvant (WPI-CN), the spherosomes were numerous, smaller (with diameters between 17.42–34.03 µm) with an agglutination tendency (Figure 1a). The *L. casei* 431^®^ formed a compact biofilm in the structure of the scales in both variants.

**Figure 2:**
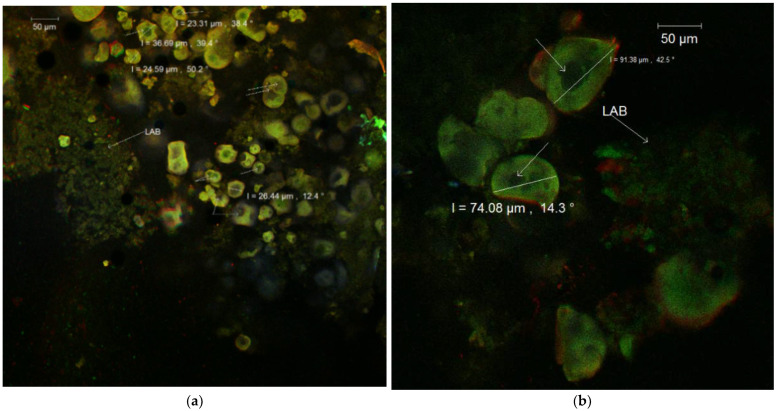
Confocal laser scanning microscopy images of co-microencapsulated extract from Cornelian cherry fruits with *L. casei* 431^®^ in whey protein isolates and casein (**a**) and whey protein isolates and inulin (**b**) stained with fluorophores.

The fluorescent labeling with Congo Red of the samples (Figure 2a,b) suggested a double encapsulation of bioactives in the spherosomes in both experimental variants.

The microstructural difference between the two powders consisted of the fact that the WPI-CN was softer, with uniform spherosomes with smaller diameters, whereas in the WPI-I, the spherosomes were bigger and reached dimensions of 74.08–91.38 µm.

### 3.4. In Vitro Digestion of Anthocyanins

**Figure 3:**
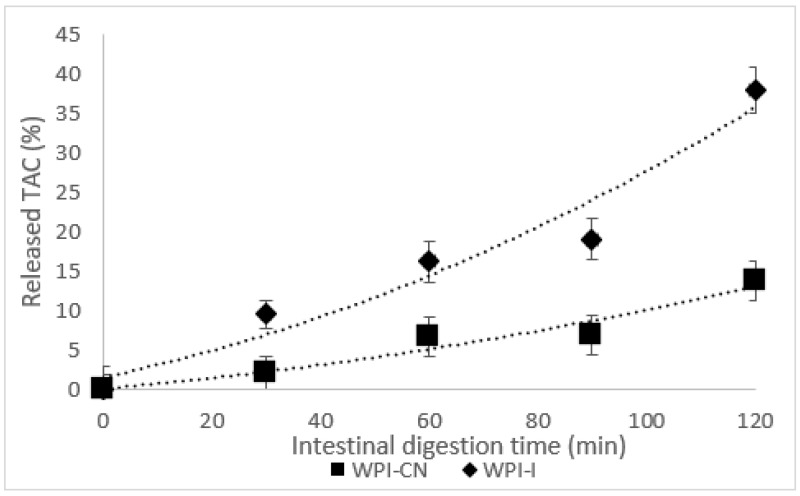
The controlled release of total anthocyanins in the simulated intestinal environment (WPI-CN—co-microencapsulated extract from Cornelian cherry fruits with *L. casei* 431^®^ in whey protein isolates and casein, WPI-I—co-microencapsulated extract from Cornelian cherry fruits with *L. casei* 431^®^ in whey protein isolates and inulin). The values represent mean ± SD (*n* = 3).

The TAC content in SGJ increased slightly with 8.75% in the WPI-CN and with 7.10% in the WPI-I, with no significant differences (*p* > 0.05) in the influence of the gastric digestion time tested (120 min) on TAC. These results highlight the protective effects of both biopolymeric matrices upon the anthocyanin compounds in SGJ. In SIJ, the controlled release of the anthocyanins was found for both of the co-microencapsulated powders (Figure 3).

The mixture of WPI-CN was less effective in releasing the anthocyanins, thus at the end of the intestinal digestion, about 14% of the anthocyanins present in the microcapsules were released. By comparison, the WPI-I matrix allowed the release of about 38% of TAC, which may be correlated with a higher absorption capacity (Figure 3). It is important to mention that both of the biopolymeric combinations had a similar protective effect for TAC in the gastric environment, whereas the WPI-I allowed a better release of the anthocyanins in the intestinal environment.

The bioaccessibility of TAC took into account the number of compounds released from the powder after the gastrointestinal digestion that could become available for the absorption into the systemic circulation [37]. In this study, the TAC bioaccessibility was calculated by dividing the TAC (mg C3R/g DM) in the digested samples after gastrointestinal digestion and the TAC (mg C3R/DM) in powder before gastrointestinal digestion. A higher bioaccessibility of TAC of 55% was found in the WPI-CN powder, when compared with 49% in the WPI-I.

### 3.5. Colorimetric Analysis

Color represents one of the most important properties underlying the quantification of the freshness of food. The colorimetric properties of the co-microencapsulated powders are shown in Table 2.

The parameter a* describes the tendency of the powder to red, which can be associated with the anthocyanins released from proanthocyanidins [38] and the formation of pigments derived from anthocyanins that stabilize the red flavilium [39]. From Table 2, it can be observed that the powders showed comparable redness and yellowness coefficients, which is in good agreement with the results for the TAC content. The L* parameter showed that the two variants of co-microencapsulated powders had a tendency to light, due to the use of the microencapsulated white materials.

### 3.6. Viability of Lactic Acid Bacteria

A critical parameter when evaluating the co-microencapsulation process of lactic acid bacteria is the guarantee of the probiotic survivability during processing stages and to keep the cells viable during storage [40]. A minimum amount of 10^6^ CFU/g of viable probiotics in the ready-to-eat product is recommended to obtain beneficial health effects [41]. The cells viability of the *L. casei* 431^®^ was tested immediately after the freeze-drying, as well as after 21 and 90 days of storage at 4 °C, in the dark. The powders showed an initial viable cells value of 9.9 Log CFU/g DM. No significant reduction in the viable counts were found after 21 days of storage (9.8 log CFU/g DM), whereas after 90 days, *L. casei* 431^®^ retained up to 97% viable cells in WPI-I and up to 93% in WPI-CN (9.18 log CFU/g DM and 9.58 log CFU/g DM, respectively). Therefore, the co-microencapsulated lactic acid bacteria cells remained viable in both powders during 90 days of storage, at 4 °C. Based on the obtained results, it is fair to conclude that the unique combination of biopolymers and bioactives from Cornelian cherry extract provided a protective environment for *L. casei* 431^®^. Neuenfeldt et al. [8] suggested that at room temperature (25 °C), the free bacteria remained viable for approximately 30 days of storage, with a cell reduction to 4.06 log CFU/g, whereas the microencapsulated *L. rhamnosus* using different blueberry extract concentrations showed viability for over 90 days, with counts above 6.0 log CFU/g.

### 3.7. Storage Stability of the Bioactive Compounds in the Co-Microencapsulated Powders

The TAC, TPC, TFC, and antioxidant activity were determined after 21 and 90 days, in order to evaluate the stability of co-microencapsulated compounds (Table 3).

From Table 3, it can be observed that TAC were the most sensitive compounds, with a significant 67% reduction in WPI-CN and 56% in WPI-I. No significant differences were found in TPC and TFC, whereas a positive correlation between the decrease in TAC and antioxidant activity was found for both samples.

The obtained results allowed the use of the first order kinetic model for the estimation of the half-life for anthocyanins content and antioxidant activity in the powders. The *t*_1/2_ values for the powders were significantly different, with 66.88 ± 1.67 days for WPI-CN and 83.60 ± 2.46 days for WPI-I, suggesting a higher stability of anthocyanins in the WPI-I matrices. Concomitantly, the *t*_1/2_ for the antioxidant activity were of 752.43 ± 2.56 days and 3009.75 ± 4.57 days, respectively, suggesting an excellent stability of biologically active compounds in the two experimental variants. The results obtained are consistent with Moser et al. [39], who concluded that grape juice encapsulated with maltodextrin and soy or whey proteins had high stability during the 150 days of storage.

In addition, Azarpazhooh et al. [30] studied the degradation kinetic of TAC in a microencapsulated pomegranate peel extract under different storage conditions (temperature of 4 °C and 25 °C and relative humidity of 52 and 75%), suggesting a higher stability of TAC at lower temperature. For example, the microencapsulated TAC in a 10% maltodextrin with calcium alginate (0.1%) revealed a half time of 115.52 days at 4 °C and a relative humidity of 75%.

### 3.8. In Vitro Anti-Proliferative Activity of the Co-Microencapsulated Powders

**Figure 4:**
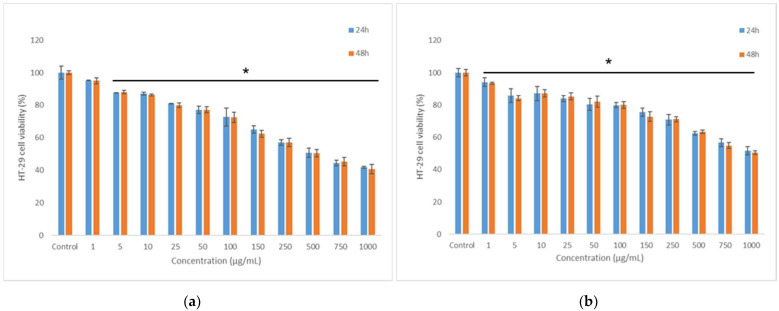
The anti-proliferative effect of co-microencapsulated powders containing *L. casei* 431^®^ in whey protein isolates and inulin (**a**) and in whey protein isolates and casein (**b**) on human intestinal cells HT-29 assessed by NR method at 24 and 48 h. The results were expressed as percent relative to the control culture (untreated), considered 100% viable. The values represent mean ± SD (*n* = 3), * *p* < 0.05, compared to control.

The anti-proliferative effect of the co-microencapsulated powders was assessed on the human intestinal cells (HT-29) by NR assay. The obtained data indicated an anti-proliferative effect of both powders, in a concentration-dependent manner (Figure 4).

**Figure 5:**
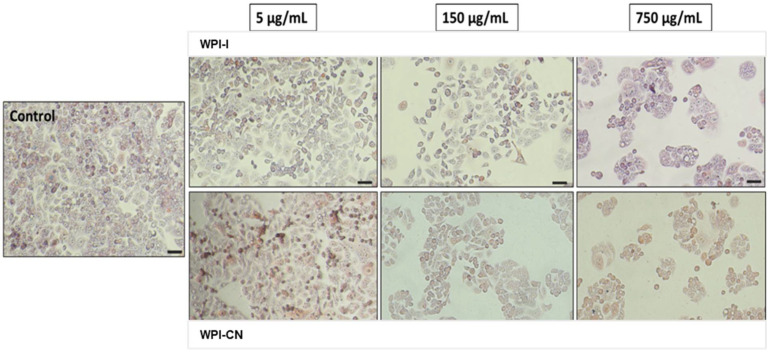
Selection of light micrographs showing the cell morphology of HT-29 tumoral cells cultivated in the presence of different concentrations of powders, for 48 h. Scale bar = 50 µm.

The effect of powders on cell viability decreased under 80% at concentrations higher than 100 µg/mL. The EC50 values were calculated in the range of 750–1000 µg/mL, with WPI-I powder having the most prominent anti-proliferative activity. Microscopy investigation of the tumoral cells morphology confirmed the NR quantitative data (Figure 5). The micrographs showed a concentration-dependent decrease of the cell density for both variants of co-microencapsulated powders. Even though treated cells maintained their normal aggregative phenotype, the cell viability and proliferation were severely affected, at concentrations higher than 250 and 750 µg/mL of WPI-I and WPI-CN variants, respectively.

Previous studies have shown the dose-dependent anti-tumoral effect of CM juice through the inhibitory effect on the viability of various cancer cell lines (Hep-G2, Caco-2, HT-29, CT-26, MCF-7), including HT-29 human colon cancer cells [42]. Recently, the anti-proliferative effect was found to be correlated with the polyphenols content and iridoid presence, as a major monoterpenoid constituent of CM [43]. For example, Blagojević et al. [43] suggested that different CM fruits genotypes exhibited high antioxidant capacity and anti-proliferative effect on HT-29 cells. However, these authors reported significant lower inhibitory concentrations in the range of 9.14 mg/mL to 13.97 mg/mL, as a function of genotype. The bioactives from CM extract considered as major contributors to anti-proliferative activity were delphinidin and cyanidin galactosides, loganic acid, and sweroside [43]. The customized designs used in our study allowed enhancing the anti-proliferative activity of the CM extracts, with a potential to inhibit HT-29 cell viability with 50% in the range of 500–1000 µg/mL.

### 3.9. In Vitro Cytocompatibility of the Co-Microencapsulated Powders

**Figure 6:**
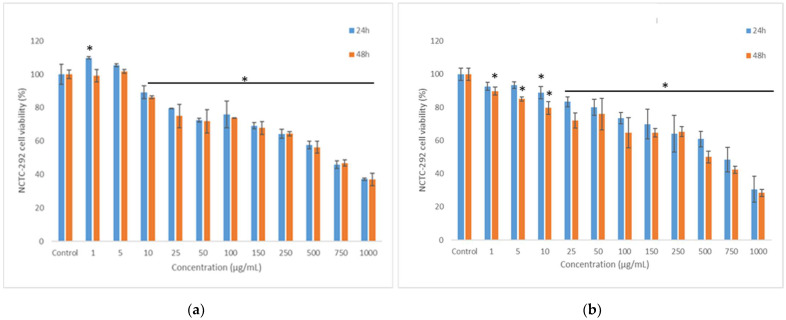
Cell viability of L929 fibroblasts cultivated in the presence of co-microencapsulated extract from cornelian cherry fruits with *L. casei* 431^®^ in whey protein isolates and inulin (**a**) and in whey protein isolates and casein (**b**) for 24 h and 48 h, respectively, determined by NR assay. The results were expressed as percent relative to the untreated culture (control), considered 100% viable. The values represent mean ± SD (*n* = 3), * *p* < 0.05, compared to control.

Cell viability was evaluated in L929 fibroblast cell culture by NR assay. The results showed that both co-microencapsulates variants were cytocompatible in a concentration-dependent manner (Figure 6). From Figure 6, it can be seen that cell viability values after 48 h of cultivation were higher than 80% at powders’ concentrations ranging from 1 μg/mL to 25 μg/mL for WPI-I variant (Figure 6a) and from 1 μg/mL to 10 μg/mL for WPI-CN variant (Figure 6b). Additionally, WPI-I variant stimulated the cell proliferation at low concentrations, ranging from 1 to 5 μg/mL, after 24 h of treatment. At concentrations between 50 and 250 μg/mL, the WPI-I variant induced a decrease of cell viability (60–80% values), with a significant decrease down to 35% at higher concentration (750—1000 μg/mL).

A wider concentration range between 1–50 μg/mL was found for the WPI-CN variant, suggesting a good cyto-compatibility (cell viability >80%). For the WPI-CN variant, a moderate cytotoxicity (60–80% cell viability) was found at concentration range of 100–250 μg/mL, after 48 h of cultivation and even 500 μg/mL at 24 h of cultivation. At higher concentrations of WPI-CN powder, the cell viability decreased down to 30%.

**Figure 7:**
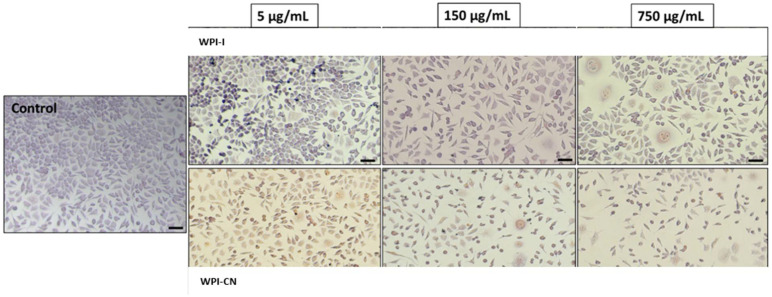
Selection of light micrographs showing the cell morphology of L929 cells cultivated in the presence of different concentrations of powders, for 48 h. Scale bar = 50 µm.

Light microscopy images showed that L929 cells cultivated in the presence of co-microencapsulated variants maintained their normal fusiform phenotype, specific for fibroblast cells, similar to the control culture, at low concentrations ranging between 1 and 50 µg/mL (Figure 7).

The cells were homogeneously distributed on the surface and their density was comparable to the control culture. At concentrations of 50–250 μg/mL, the cell density slightly decreased, while at higher concentrations, some structural modifications of cells, such as vesicle formation were observed in cells treated with both variants. Thus, these qualitative observations of light microscopy confirmed the NR quantitative data of cytocompatibility.

Popović et al. [44] used freeze-dried CM fruit to obtain free and microencapsulated extracts by using inclusion complexes with β-cyclodextrin. These authors tested the cytotoxic profile of β-cyclodextrin, Cornelian cherry water/ethanol extract, and β-cyclodextrin encapsulate of 50% ethanolic-water extract on HT-29 cell lines. A significant higher cytotoxic profile ranging from 312.5 to 2500 μg/mL was suggested, with a uniform cytotoxic response and low dose-dependent increase of cytotoxic activity in evaluated concentration range for the microencapsulated variant.

However, when considering the anti-proliferative and cytotoxic effects of the powders in different food, pharmaceutical, and nutraceutical applications, the safety dose should be further investigated in appropriate toxicity studies.

## 4. Conclusions

In this study, the prebiotic effect of Cornelian cherry fruits extract on the *Lacticaseibacillus casei* (*L. casei*) 431^®^ was tested through the formulation of two variants of co-microencapsulation based on whey proteins isolate with casein and inulin, by a complex coacervation and freeze-drying. Both powders revealed a good retention of anthocyanins, with a microencapsulation efficiency of 78–79% for anthocyanins and 90% for lactic acid bacteria. Two pink-reddish powders were obtained, with a significant content of bioactive compounds and viable cells, of 10^9^ CFU/g DW, with different structure and morphologies particularities. The design of the synbiotic delivery systems based on whey protein isolate and casein led to soft microparticles, with uniform spherosomes and small diameters, whereas the unique combinations of biopolymers allowed a double microencapsulation, therefore explaining the in vitro resistance of anthocyanins, especially in the acidic environment. The in vitro digestion revealed the protective effect of the encapsulating matrices on the anthocyanins in the gastric environment, whereas the combination of whey protein isolates and inulin was more effective for anthocyanins’ release in the intestinal simulated juice. During storage, anthocyanins were more sensitive to the environmental conditions, while a clear cell viability of at least 9 Log CFU/g DW in both powders was observed. The inulin-containing variant showed the most potent anti-proliferative effect, whereas both powders were cytocompatible, but to a different extent. In conclusion, our results demonstrated the potential to develop multifunctional ingredients, provided by both the bioactive compounds from plant sources, Cornelian cherries, and the probiotic lactic acid bacteria, for different applications, such as adding or using these types of powders in nutraceuticals or in food products.

## Figures and Tables

**Table 1:** Co-microencapsulation efficiency, phytochemical content, and *L. casei* 431^®^ content of the co-microencapsulated powders.

Analyzed Parameter	WPI-CN	WPI-I
Anthocyanins’ co-microencapsulation efficiency (%)	77.97 ± 0.57	79.03 ± 0.72
*Lactobacillus casei* 431^®^ co-microencapsulation efficiency (%)	90.03 ± 0.56 ^a^	90.07 ± 0.34 ^a^
Total monomeric anthocyanins content (mg C3R/g DM)	32.14 ± 0.97 ^a^	32.29 ± 0.26 ^a^
Total polyphenolic content (mg GAE/g DM)	9.67 ± 0.12 ^a^	9.79 ± 0.15 ^a^
Total flavonoids content (mg CE/g DM)	5.59 ± 0.51 ^a^	5.94 ± 0.24 ^a^
Antioxidant activity (mMol Trolox/g DM)	50.05 ± 0.94 ^a^	47.62 ± 1.43 ^b^
*Lactobacillus casei* 431^®^ (CFU/g DM)	8.02 × 10^9 a^	8.10 × 10^9 a^

WPI-CN—Co-microencapsulated extract from Cornelian cherry fruits with *L. casei* 431^®^ in whey protein isolates and casein. WPI-I—Co-microencapsulated extract from Cornelian cherry fruits with *L. casei* 431^®^ in whey protein isolates and inulin. For each tested parameter and powder, values that are on the same row that do not have the same lowercase letters ((^a^) and (^b^)) are statistically different at *p* < 0.05 based on the Tukey method and the 95% confidence interval.

**Table 2:** Colorimetric analysis of the powders.

Variants	L*	a*	b*	c*	h*	ΔE
WPI-CN	37.17 ± 0.24 ^a^	21.45 ± 0.18 ^a^	6.67 ± 0.01 ^a^	22.47 ± 0.17 ^a^	0.30 ± 0.01 ^a^	43.43 ± 0.29 ^a^
WPI-I	35.35 ± 0.06 ^b^	21.30 ± 0.12 ^a^	6.00 ± 0.02 ^b^	22.13 ± 0.12 ^a^	0.27 ± 0.01 ^b^	41.71 ± 0.11 ^b^

WPI-CN—Co-microencapsulated extract from Cornelian cherry fruits with *L. casei* 431^®^ in whey protein isolates and casein. WPI-I—Co-microencapsulated extract from Cornelian cherry fruits with *L. casei* 431^®^ in whey protein isolates and inulin. For each parameter tested, the values that are on the same column that do not have the same lowercase letters ((^a^) and (^b^)) are statistically different at *p* < 0.05 based on the Tukey method and the 95% confidence interval.

**Table 3:** Storage stability of bioactive in co-microencapsulated variants during storage.

Variants	Storage (Days)	Antioxidant Activity (mMol/g DM)	TPC (mg GAE/g DM)	TFC (mg CE/g DM)	TAC (mg C3R/g DM)
WPI-CN	0	50.05 ± 0.94 ^a^	9.67 ± 0.12 ^a^	5.59 ± 0.51 ^a^	32.14 ± 0.97 ^a^
	21	49.73 ± 2.35 ^b^	9.65 ± 0.07 ^a^	5.65 ± 0.31 ^a^	14.62 ± 1.12 ^b^
	90	46.33 ± 1.01 ^a^	9.57 ± 0.08 ^a^	5.85 ± 0.65 ^a^	10.64 ± 0.66 ^b^
WPI-I	0	47.62 ± 1.43 ^b^	9.79 ± 0.15 ^a^	5.94 ± 0.24 ^a^	32.29 ± 0.26 ^a^
	21	46.03 ± 0.24 ^a^	9.75 ± 0.02 ^a^	5.81 ± 0.41 ^a^	17.26 ± 0.81 ^a^
	90	45.87 ± 1.11 ^a^	9.54 ± 0.02 ^a^	5.67 ± 0.33 ^a^	13.35 ± 0.37 ^a^

WPI-CN—Co-microencapsulated extract from Cornelian cherry fruits with *L. casei* 431*^®^* in whey protein isolates and casein. WPI-I—Co-microencapsulated extract from Cornelian cherry fruits with *L. casei* 431^®^ in whey protein isolates and inulin. Values that are on the same row that do not have the same lowercase letters ((^a^) and (^b^)) are statistically different at *p* < 0.05 based on the Tukey method and the 95% confidence interval.

## Data Availability

Data will be made available on reasonable request.
